# Advance Age Pregnancy in a Tertiary Care Centre: A Descriptive Cross-sectional Study

**DOI:** 10.31729/jnma.6008

**Published:** 2021-04-30

**Authors:** Vibha Mahato, Pravin Shrestha, Smita Shrestha

**Affiliations:** 1 Department of Obstetrics and Gynaecology, Manipal College of Medical Sciences, Pokhara, Nepal; 2 Nobel College, Sinamangal, Kathmandu, Nepal

**Keywords:** *advanced*, *age*, *maternal*, *outcome*, *perinatal*

## Abstract

**Introduction:**

Women who conceive at advance age are at risk of pregnancy complications and adverse foetal outcome. This study aims to find out the prevalence of pregnancy at advance age in a teaching hospital.

**Methods:**

A descriptive cross-sectional study was conducted between October 2019 to August 2020 at department of obstetrics and gynaecology of a tertiary care centre of Nepal, after obtaining ethical clearance from Institutional Review Committee (dated 03/09/2019 with ref no. 266) and informed consent from patient. Convenience sampling was done. All the patient who were ≥35 years and >28 weeks of gestation without any chronic illness were selected. Data and descriptive analysis were done using Statistical Package for the Social Sciences version 16. Point estimate at 95% Confidence Interval was calculated along with frequency and percentage for binary data.

**Results:**

Women aged 35 years and above constituted 104 (5.73%) of the total deliveries of study period. Most were multigravida 72 (69.23%) and 23 (22.12%) had preterm delivery. Rate of caesarean section were higher in advance maternal age 69 (66.35%). Maternal complications such as Hypertensive disorder of pregnancy 9 (8.65%), and mal-presentation 15 (14.42%) were higher among them. Perinatal outcome in form of low birth weight 9 (8.65%) and perinatal death 5 (4.80%) were increased in those women.

**Conclusions:**

From this study, it can be concluded that prevalence of advanced age at pregnancy was lower than study done in developed country but it was similar to study in India and is increasing in Nepal.

## INTRODUCTION

Advanced maternal age is defined as 35 or more years at the time of delivery.^1^ A study done in 29 countries revealed average prevalence of advanced maternal age was 12.3% and 2.8% in Nepal.^2^

With increasing maternal age pregnancy may be complicated by hypertensive disorder of pregnancy, gestational diabetes mellitus (GDM), and antepartum haemorrhage. There is higher incidence of caesarean section, and postpartum haemorrhage and is a risk factor for stillbirth. Neonates are affected due to prematurity, low birth weight, Intra Uterine Growth Restriction, and foetal distress, which increase rates of Neonatal Intensive Care Unit admission. These increased risks appeared to be independent of maternal co-morbidities.^3,4^

Although pregnancy with advanced age has also been increasing in developing countries like ours, limited study has been conducted to identify adverse outcome of pregnancy at advance age.

Hence, we aimed to find out the prevalence of advance maternal age at pregnancy in a tertiary hospital.

## METHODS

A descriptive cross-sectional study was conducted in the Department of Obstetrics and Gynaecology of Manipal Teaching Hospital, Pokhara, Nepal between October 2019 to August 2020. This study was conducted after approval of Institutional Review Committee (dated 03/09/2019 with ref no. 266) and consent of patient or patient's relatives. Convenience sampling was used to collect the data. All the patient who were ≥35 years and >28 weeks of gestation without any chronic illness were included. Exclusion criteria were all pregnant woman below the age of 35 years, less than 28 weeks of gestation, multiple pregnancy, and patients with chronic illness such as renal failure, severe cardiac disease, chronic liver disease and chronic lung disease. Sample size was calculated using the formula,

$$\begin{array}{ll} \text{n} & \text{= }{\text{Z}}^{\text{2}}\times \text{p}\times (1-\text{p)}/{\text{e}}^{\text{2}}\\ & \text{= }1.96^{\text{2}}\times 0.5\times 0.5/\text{0}{\text{.03}}^{\text{2}}\\ & \text{= }1067 \end{array}$$

where,

n = minimum required sample size
Z = 1.96 at 95% Confidence Interval
p = prevalence of advance age pregancy, 50%
e = margin of error, 3%.

Adding a 10% non-response rate, the sample size was 1174. But we collected data from 1813 patients.

All patients were analysed for maternal outcome such as, pregnancy induced hypertension, gestational diabetes mellitus, placenta Previa, Premature Rupture of Membranes (PROM), malpresentation, oligohydramnios and postpartum haemorrhage. Perinatal outcome analysed as, preterm birth, Intrauterine Growth Retardation (IUGR), birth weight, perinatal deaths, and congenital anomalies. Information related to patient's general characteristics (age at the time of delivery, gravida, and gestational age), complication during pregnancy, mode of delivery and perinatal outcome was recorded in a predesigned pro forma and the data was tabulated in excel sheet.

The recorded data were analysed statistically using Statistical Package for Social sciences (SPSS) version 16.

## RESULTS

Out of 1813 deliveries conducted during the study period, out of them 104 were above the age of 35 years at the time of admission. The overall prevalence of advanced maternal age at pregnancy was 104 (5.73%).

Among them, 91 (87.50%) were of advance maternal age (35-39 years) and 13 (12.50%) were of very advance age (≥40 years).

It was observed that multigravida 72 (69.23%) were more common in advanced maternal age. It appears that, majority had their first child before 35 years old and had delayed the second and subsequent pregnancy. In our study, rate of caesarean section 69 (66.35%) was higher in women 35 years and older as compared to vaginal delivery 32 (30.77%) (Table 1).

**Table 1:** Demographic characteristics and health variable of above 35 years patients.

Variables	n (%)
Age	
35-39 years	91 (87.50)
≥40 years	13 (12.50)
Gravida	
Multigravida	72 (69.23)
Grand multigravida	19 (18.26)
Prim gravida	13 (12.50)
Gestational age at delivery	
≥37	81 (77.88)
<37	23 (22.12)
Mode of delivery	
Caesarean section	69 (66.35)
Vaginal delivery	32 (30.77)
Instrumental delivery	3 (2.89)

Women of age 35 and above were found to have higher preterm birth as a pregnancy complication i.e. 23 (22.12%) followed by mal-presentation 15 (14.42%), hypertensive disorder 9 (8.65%), premature rupture of membrane 7 (6.73%), postpartum haemorrhage 5 (4.80%), gestational diabetes mellitus 4 (3.84%) and antepartum haemorrhage 4 (3.84%) (Table 2).

**Table 2:** Pregnancy complication in advance maternal age.

Pregnancy complications	n (%)
Preterm birth	23 (22.12)
Malpresentation	15 (14.42)
Hypertensive disorder in pregnancy	9 (8.65)
Premature Rupture of Membrane	7 (6.73)
Postpartum haemorrhage	5 (4.80)
Gestational Diabetes Mellitus	4 (3.84)
Antepartum haemorrhage	4 (3.84)

Nine babies were low birth weight 9 (8.65%). Therewere 5 (4.80%) perinatal deaths inwomen of age 35 and above. Only 1 (0.96%) patient had congenital anomaly in the form of cleft lip and palate (Table 3).

**Table 3:** Perinatal outcome in advance maternal age

Perinatal Outcome	n (%)
Low birth weight	9 (8.65)
Perinatal Death	5 (4.80)
Intrauterine Growth Restriction	2 (1.92)
Congenital anomalies	1 (0.96)

## DISCUSSION

Pregnancy at 35 years or more is continuously rising. In Canada, there has been an increase in the percentage of births from women aged between 35-39 years, ranging from 4.7% in 1982 to 14.1% in 2002.^4^ The incidence of pregnancy at advanced maternal age in US was reported as 23% in 2014 and 4.5% from India by Ritu, et al.^1,3^ Prevalence of advance maternal age in our study (5.7%) is much lower than developed country but was comparable with the study done in India.^1,3^ According to a multi-country data of 2014 The overall prevalence of 29 countries was 12.3% and the prevalence of advanced maternal age was 2.8% in Nepal. ^2^ In our study prevalence was 5.7% which was more as compared to multi-country data of Nepal but was below the world average.

It was observed that 87.50% were age 35-39 years and 12.50% were of age ≥40 years compared to study done by Rajput N, et al. where group of 35 year to 39 years more (89.93%) and above 40 year was much less than our study (2.08%).^5^

In our study we observed 69.23 % were multigravida and 18.26% were grand multi gravida. This result was comparable to the study done by Rajput N, et al. where 71% were multigravida and 22.22% were grand multigravida.^5^ This may be because of lack of education of contraception and desire of male baby in our area.

There was higher rate of caesarean section (66.35%) was observed in women of age 35 or more. we found that common indication for caesarean section was previous caesarean section (45.24%) followed by foetal distress (11.16%). Our result were much higher than the studies done by Ogawa et al and Ritu et al where rate of caesarean section were only 32.1% and 42.% respectively.^1,6^

Kahveci, et al. reported 8.65% hypertensive disorder in pregnancy with advance maternal age group which is similar to our study (9.47%).^7^

In advance maternal age 14.42% were malpresentation. This observation was similar to study done by H.-C. Lin, et al. (13.80%).^8^ In this study common malpresentation observed was breech. Pre-labour rupture of membranes and antepartum haemorrhage were also more common in advance maternal age (6.73% and 3.84% respectively).^8^ These result were comparable with the study done by Ritu et al. where pre-labour rupture of membrane was 7.80% and antepartum haemorrhage was more than our study (6.50%).^9^ Preterm birth (22.12%) was found to be the common complication of advance maternal age higher to study done by Pawde et al. were 17% were preterm.^9^

Maternal complication such as PPROM, preeclampsia, antepartum haemorrhage were the major causes of preterm birth in our study. We also found that 4.80% of advance age mother had post-partum haemorrhage. Mehari, et al. and Radon-Pokracka, et al. showed similar result were 5.2% and 4.3% respectively had post-partum hemorrhage.^10, 11^ Lower contraction potential and decreased oxytocin receptor of uterus in elderly women might be the result of PPH.^12^

We also observed that 8.65% of babies were of low birth weight. Our results are similar to study done by Radon-Pokracka, et al. where low birth weight in advance maternal age was 10.7%.^11^ Low birth weight may be due to age-related changes in the uterine vasculature, poorer placental perfusion. In our study five women of 35 years and more had perinatal death (4.80%) where two had intrauterine foetal death, one had still birth and two were neonatal death. Similarly, Lean SC, et al. also reported increased rate of perinatal death (4.70%).^13^ Antenatal complications, such as preeclampsia, antepartum haemorrhage and preterm birth can be the reason for perinatal death in our study.

Congenital anomalies (0.96%) was observed in our study in women of 35 years and more. This is similar to study done by Pawde et al (0.95%).^9^

The main limitation of our study was single centre population and lack of data on race/ethnicity, socioeconomic status and BMI which also contribute to adverse pregnancy outcome. Our study also failed to collect information about reason for delayed pregnancy. This failure to examine the population of different centre and reason for delayed pregnancy may be addressed in future studies.

## CONCLUSIONS

Prevalence of advance maternal age is increasing with increasing time. This study has shown that pregnancies over 35 years of age are high-risk pregnancies and have higher incidence of hypertensive disorder of pregnancies, premature rupture of membrane and malpresentation. Caesarean delivery was increased in those mothers. Along these advanced maternal age pregnancy was also found to be a major risk factor for preterm delivery, low birth-weight and perinatal death. So, they should be advised for frequent antenatal visits and increased maternal and foetal surveillance should be done to ensure a better maternal and perinatal outcome.
